# Chloroform Water

**Published:** 1883-03

**Authors:** 


### Chloroform Water.

This is prepared by mixing three parts of water with one of chloroform, shaking the mixture well, and letting it stand for some time. The excess of chloroform is then removed by decantation, or is drawn off with a syphon; the water thus obtained contains nine parts of chloroform to one hundred parts of water. This is a good solvent for chlorate of potash, bicarbonate of soda, salicylate of soda. Chloroform water, as thus obtained, is said to be the best preparation wherever the internal use of chloroform is indicated.

Chloroform water is very agreeable to the taste, and leaves in the mouth a sensation of coolness, lasting for a few minutes after the water is swallowed. This water is well adapted as a vehicle for medicines having a disagreeable taste, its own peculiar taste generally acting as a complete disguise. For its local action this preparation can be benificially used in many affections of the mouth, gums, palate and pharynx. Its action on the stomach varies according to the circumstances under which it is taken. Taken with wine it increases its stimulant properties. It is efficient in combating digestive disturbances, acting as a local sedative as when in contact with the mucous surface of the mouth. REVISTA DE MEDICINA.

